# Supplementary material for: Low PCSK-9 levels Are Associated with Favorable Neurologic Function after Resuscitation from out of Hospital Cardiac Arrest
Source: J Clin Med. 2020 Aug 11;9(8):2606. doi: 10.3390/jcm9082606 (PMC7465607; doi:10.3390/jcm9082606)
Supplement: Supplementary file 1 [file jcm-09-02606-s001.pdf]

SUPPLEMENT

Table S1. Logistic regression: outcome: 30-day neurologic function defined by Cerebral Performance Categories (CPC) 1 and 2.

Favorable neurological outcome CPC 1 and 2	Odds ratio (95% confidence intervals)	*p*-value
PCSK-9 ≥ 165mg/dL	4.72 (1.76–12.66)	0.002
PCSK-9 ≥ 165mg/dL	3.67 (1.32–10.16)	0.013
C-reactive protein > 0.5mg/dL at admission	2.48 (0.81–7.65)	0.114
PCSK-9 ≥ 165mg/dL	4.38 (1.61–11.87)	0.004
Sex	0.45 (0.14–1.51)	0.20
PCSK-9 ≥ 165mg/dL	4.46 (1.61–12.38)	0.004
Age (years)	1.04 (1.00–1.08)	0.041
PCSK-9 ≥ 165mg/dL	4.73 (1.48–15.18)	0.009
No-flow time (min)	1.13 (0.90–1.42)	0.293
PCSK-9 ≥ 165mg/dL	5.199 (1.84–14.71)	0.002
Initial shockable rhythm	0.26 (0.08–0.89)	0.032

PCSK-9, proprotein convertase subtilisin/kexin type 9.

Table S2. Baseline Characteristics: Categorical data are presented as counts and percentages, continuous data as medians and interquartile ranges (IQRs).

**Baseline Characteristics.**	**Total (*n* = 79)**	**PCSK9-levels (ng/mL)**		***p***
		**<180 (*n* = 32)**	**≥180 (*n* = 47)**	
Gender, male *n* (%)	61 (77)	27 (84)	34 (72)	.214
Age, years (IQR)	59 (46–69)	56 (45–65)	59 (51–70)	.169
Concomitant diseases, *n* (%)				
Hyperlipidemia	20 (25)	6 (19)	14 (30)	.271
DM II	16 (20)	7 (22)	9 (19)	.769
Coronary artery disease	16 (20)	7 (22)	9 (19)	.769
Hypertension	31 (39)	13 (41)	18 (38)	.836
Smoker	26 (33)	10 (31)	16 (34)	.797
COPD	8 (10)	3 (9)	5 (11)	.560
PAD	6 (8)	0 (0)	6 (13)	.037
Statine-use, *n* (%)	14 (18)	3 (9)	11 (23)	.111
CPC 1/2, *n (%)*	36 (46)	20 (63)	16 (34)	.013
Witnessed, *n* (%)	64 (81)	25 (78)	39 (83)	.454
BLS, *n* (%)	55 (70)	22 (69)	33 (70)	.663
Initial shockable rhythm, *n* (%)	59 (75)	25 (78)	34 (72)	.564
Time to sustained ROSC, min (IQR)	25 (17–43)	23 (14–43)	28 (20–42)	.205
No-flow time, min (IQR)	0.0 (0.0–3.0)	0.0 (0.0–2.1)	0.0 (0.0–3.0)	.808
Low-flow time, min (IQR)	25.0 (16.0–40.5)	22 (11–41)	25 (20–41)	.262
30-day survival, *n* (%)	53 (67)	27 (84)	26 (55)	.007
Core body temperature (admission), °C (IQR)	35.3 (34.8–35.8)	35.5 (34.9–35.9)	35.1 (34.7–35.6)	.223
Blood gas values (admission)				
pH (IQR)	7.16 (7.04–7.25)	7.16 (7.00–7.22)	7.16 (7.05–7,25)	.996
Lactate, mmol/L (IQR)	7.6 (5.2–10.0)	7.25 (4.8–10.1)	7.7 (5.9–9.9)	.577
Laboratory values (admission)				
Hemoglobin, g/dL (IQR)	13.9 (12.4–15.3)	14.1 (12.5–15.3)	13.8 (12.3–15.3)	.809
Total Platelet count, G/L (IQR)	205 (169–245)	226 (175–252)	200 (163–245)	.419
Leukocytes, G/L (IQR)	14.3 (10.5–18.9)	14.6 (9.1–18.9)	14.2 (10.8–19.1)	.822

Troponin-T, ng/L (IQR)	64 (32–244)	113 (37–367)	53 (27–195)	.187
Prothrombin-time, % (IQR)	77 (65–91)	72 (59–83)	81 (66–91)	.162
TG, mg/dL (IQR)	125 (81–173)	111 (82–159)	136 (78–180)	.221
Creatinin, mg/dl (IQR)	1.1 (0.9–1.3)	1.2 (0.9–1.3)	1.1 (0.9–1.3)	.830
Albumin, g/L (IQR)	38 (34–40)	39 (36–41)	38 (33–40)	.102
Cholinesterase, kU/L (IQR)	6.70 (5.4–8.0)	6.76 (4.96–7.96)	6.71 (5.64–8.32)	.371
Total cholesterol, mg/dL (IQR)	163 (133–201)	158 (138–182)	168 (128–212)	.775
ASAT (GOT), U/L (IQR)	135 (78–226)	136 (78–180)	135 (80–269)	.708
ALAT (GPT), U/L (IQR)	112 (56–200)	106 (64–166)	129 (54–210)	.526
Laboratory values				
PCSK-9 (admission), ng/ml (IQR)	193 (145–239)	128 (118–153)	226 (201–270)	.000
PCSK-9 (12h), ng/ml (IQR)	210 (151–267)	161 (130–223)	240 (203–303)	.000
PCSK-9 (24h), ng/ml (IQR)	187 (198–457)	150 (114–220)	202 (174–247)	.005
NSE (admission), µg/L(IQR)	575 (575–575)	*n*.a	575 (575–575)	*n*.a
NSE (24h), µg/L (IQR)	33.1 (21.4–54.4)	30.2 (20.9–43.1)	36.6 (21.8–57.6)	.374
S-100 (admission), µg/L (IQR)	3.78 (3.78–3.78)	*n*.a	3.78 (3.78–3.78)	*n*.a
S-100 (24h), µg/L (IQR)	0.15 (0.09–0.36)	0.11 (0.08–0.16)	0.19 (0.11–0.57	.005
CRP (admission), mg/dL (IQR)	0.2 (0.1–0.6)	0.2 (0.1–0.3)	0.3 (0.1–0.9)	.041
CRP (12h), mg/dl (IQR)	0.99 (0.6–1.6)	0.9 (0.5–1.1)	1.3 (0.7–2.2)	.035
CRP (24h), mg/dl (IQR)	4.2 (2.8–6.1)	3.8 (2.7–5.6)	4.3 (2.8–8.0)	.384

CPC, cerebral performance category; DM II, diabetes mellitus II; COPD, chronic obstructive pulmonary disease; PAD, peripheral arterial disease; BLS, basic life support; ROSC, return of spontaneous circulation; PCSK-9, proprotein convertase subtilisin/kexin type 9; TG, triglycerides; ASAT, aspartate aminotransferase; GOT, glutamate oxalacetate transaminase; ALAT, alanine aminotransferase; GPT, glutamate pyruvate transaminase; CRP, C-reactive protein; NSE, neuron-specific enolase.
